# Ketogenic diet modifies the gut microbiota in a murine model of autism spectrum disorder

**DOI:** 10.1186/s13229-016-0099-3

**Published:** 2016-09-01

**Authors:** Christopher Newell, Marc R. Bomhof, Raylene A. Reimer, Dustin S. Hittel, Jong M. Rho, Jane Shearer

**Affiliations:** 1 Department of Biochemistry and Molecular Biology, Cumming School of Medicine, University of Calgary, 3330 Hospital Dr. NW., Calgary, Alberta T2N 4N1 Canada; 2 Faculty of Kinesiology, University of Calgary, Calgary, Alberta Canada; 3 Department of Pediatrics, Cumming School of Medicine, University of Calgary, Calgary, Alberta Canada; 4 Department of Clinical Neurosciences, Cumming School of Medicine, University of Calgary, Calgary, Alberta Canada; 5 Department of Physiology & Pharmacology, Cumming School of Medicine, University of Calgary, Calgary, Alberta Canada

**Keywords:** Ketogenic diet, Gut microbiome, Autism spectrum disorder, BTBR mouse

## Abstract

**Background:**

Gastrointestinal dysfunction and gut microbial composition disturbances have been widely reported in autism spectrum disorder (ASD). This study examines whether gut microbiome disturbances are present in the BTBR^T + tf/j^ (BTBR) mouse model of ASD and if the ketogenic diet, a diet previously shown to elicit therapeutic benefit in this mouse model, is capable of altering the profile.

**Findings:**

Juvenile male C57BL/6 (B6) and BTBR mice were fed a standard chow (CH, 13 % kcal fat) or ketogenic diet (KD, 75 % kcal fat) for 10–14 days. Following diets, fecal and cecal samples were collected for analysis. Main findings are as follows: (1) gut microbiota compositions of cecal and fecal samples were altered in BTBR compared to control mice, indicating that this model may be of utility in understanding gut-brain interactions in ASD; (2) KD consumption caused an anti-microbial-like effect by significantly decreasing total host bacterial abundance in cecal and fecal matter; (3) specific to BTBR animals, the KD counteracted the common ASD phenotype of a low Firmicutes to Bacteroidetes ratio in both sample types; and (4) the KD reversed elevated *Akkermansia muciniphila* content in the cecal and fecal matter of BTBR animals.

**Conclusions:**

Results indicate that consumption of a KD likely triggers reductions in total gut microbial counts and compositional remodeling in the BTBR mouse. These findings may explain, in part, the ability of a KD to mitigate some of the neurological symptoms associated with ASD in an animal model.

**Electronic supplementary material:**

The online version of this article (doi:10.1186/s13229-016-0099-3) contains supplementary material, which is available to authorized users.

## Introduction

Autism spectrum disorder (ASD) encompasses several neurodevelopmental disorders in which seizures and gastrointestinal (GI) dysfunction exist as symptoms [1, 2]. Residing within the GI tract, the gut microbiome is a vastly diverse ecosystem comprised of trillions of bacteria and other microorganisms [3]. Yielding a metabolic capacity of ~100-fold greater than the human liver, these bacteria have been implicated in altering host metabolism and immune function [3, 4]. Recent research has also demonstrated alterations in the microbial profile of both a valproic acid-exposed animal model of ASD [5] and patients with ASD compared to controls [1, 2].

The BTBR^T + tf/j^ (BTBR) mouse mimics the behavioral phenotype associated with ASD, although it lacks spontaneous seizure activity [6, 7]. The ketogenic diet (KD), which has been implemented as a treatment to pharmacologically resistant epilepsy since the early twentieth century [6], has been shown to improve the core symptoms of ASD in BTBR animals [6]. This work represents the first successful use of a dietary therapy to counteract communication defects, repetitive behaviors, and impairments in sociability in a mouse model of ASD. Comprising of a high proportion of fat, adequate protein, and low carbohydrates, the KD causes a drastic shift in host metabolism by mimicking the fasting state and promoting ketone body production and utilization. Although the KD primarily impacts neural tissue [8], the impact of such radical metabolic changes on the gut microbiome has yet to be examined.

Research investigating both brain and gut function has demonstrated that both tissues have various degrees of involvement in ASD progression [5, 9]. A recent unifying theory termed the “gut-brain axis” outlines the interactions between the brain and microbiome, suggesting a possible connection in ASD [9, 10]. This research proposes that disruption to gut microbiota composition or diversity can modulate behavior and neural biochemistry. Therefore, the aim of this study was to examine the impact of a KD on the gut microbiota of a mouse model of ASD.

## Methods

### Animals and dietary interventions

All experimental protocols were in compliance with the ethical standards approved by the University of Calgary Animal Care and Use Committee. Juvenile male C57BL/6 (B6) and BTBR^T + tf/j^ mice (*n* = 21 and 25, respectively) were age-matched to 5 weeks of age before being randomly selected for implementation of a standard chow (CH, 13 % kcal fat) or ketogenic diet (KD, 75 % kcal fat; Bio-Serv F3666, Frenchtown, USA) for 10–14 days. The time point of 7 weeks of age for both B6 and BTBR mice was established to ensure both cohorts were post-pubertal in development [11]. Prior to sacrifice, the animals were housed in a humidity-controlled room with a 12-h light/dark zeitgeber cycle and were fed ad libitum. Following 10–14 days of dietary intervention, the mice were sacrificed by cervical dislocation. At the time of sacrifice, the animals were 7 weeks of age.

### Behavioral analysis

Previously published work on the BTBR mouse has examined the impact of the KD on several behavioral measures at similar ages as the mice in the present study [12]. Briefly, self-directed repetitive behavior (self-grooming) was quantified in the three-chamber sociability test, and communication was assessed by social transmission of a food preference [6]. This data has been re-reported and is shown to provide a frame of reference for the present work on the gut microbiome in relation to the diet.

### DNA extraction and qRT-PCR analysis

Fresh fecal samples were collected prior to sacrifice and cecal contents collected posthumously. All samples were stored at −80 °C until analysis. Using ~250 mg of fecal/cecal matter, total DNA was extracted and quantified as previously described [13]. Microbial profiling was conducted using an iCycler (BioRad, Hercules, USA) as previously reported [13]. Standard curves were normalized to the copy number of 16S ribosomal RNA (rRNA) genes using reference strain genome size and 16S rRNA gene copy number values obtained from the following reference [14]. Data are expressed as 16S rRNA gene copies/mg cecal or fecal matter. Group-specific primers are shown in Additional file 1: Table S1 and referenced in previously published work [13]. All baseline animal and transformed gut microbiota data have been previously published [15], and they are included here to provide a frame of reference for the autism-related data. Previously published work examines the mathematical relationship between specific gut microbes and serum metabolomics and not ASD.

### Statistical analysis

Statistical analysis was performed using IBM SPSS Statistics for Windows, version 20.0. Data are expressed as mean ± SEM. Differences between the genotype and diet were determined by analysis of variance (ANOVA), followed by Tukey’s post hoc test where *p* < 0.05 was considered to be significant. Microbial data was normalized and uploaded into MetaboAnalyst 3.0 for modeling using partial least squares discriminant analysis (PLS-DA). Variable importance of projection (VIP) scores were assessed in order to rank each microbial group for their degree of discrimination within the model.

## Findings

The BTBR mouse exhibits many behavioral phenotypes relevant to ASD including impaired vocalizations and social interactions [12, 16]. Previous work investigating the impact of the KD on the BTBR demonstrates the diet to improve sociability and communication of food preference while decreasing self-directed repetitive behaviors [6]. The mechanism(s) mediating these benefits are presently unknown, but many involve the gut microbiota communication. The objective of the present study was to examine the impact of a KD on the gut microbiota of a mouse model of ASD.

The major findings of this study are as follows: (1) gut microbiota composition of cecal and fecal samples were altered in BTBR compared to control mice, indicating that this model may be of utility in understanding gut-brain interactions in ASD; (2) KD consumption caused an “anti-microbial”-like effect by significantly decreasing total host bacterial abundance in cecal and fecal matter; (3) specific to BTBR animals, the KD counteracted the common ASD phenotype of a low Firmicutes to Bacteroidetes ratio in both cecal and fecal matter; and (4) the KD reversed the elevated *Akkermansia muciniphila* content in the cecal and fecal matter of BTBR animals.

**Fig. 1:**
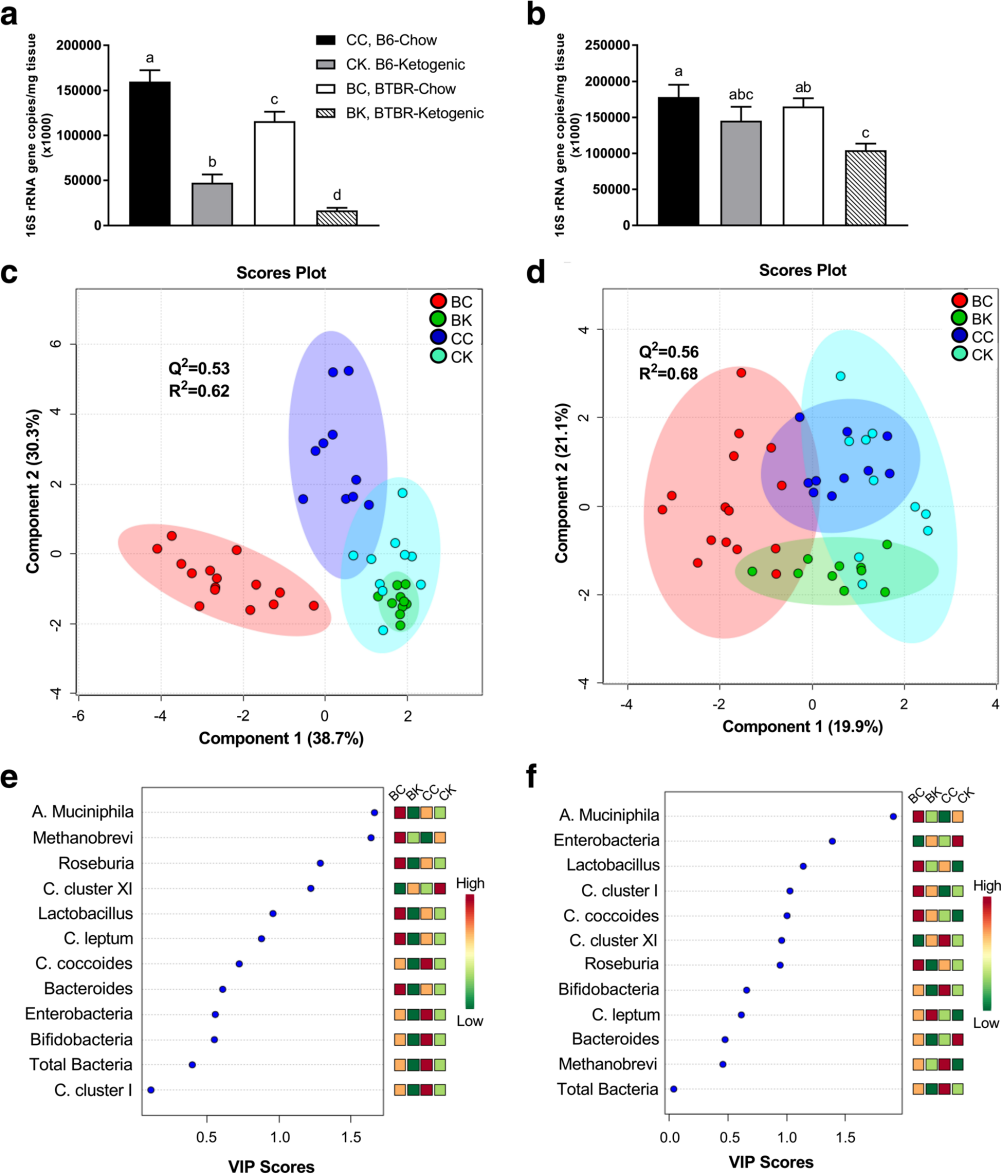
Assessment of total microbial content, partial least squares discriminant analysis (PLS-DA), and variable importance of projection (VIP) scores for relative bacterial abundance. Descriptive comparisons of total microbial content, score plots of PLS-DA, and VIP scores for both cecal and fecal matter are presented. **a** Total bacterial species composition measured from cecal matter. **b** Total bacterial species composition measured from fecal matter. **c** PLS-DA score plot for cecal matter showing model discrimination between each genotype. **d** PLS-DA score plot for fecal matter showing model discrimination between each genotype. **e** Cecal VIP plot indicating the most discriminating bacteria in descending order of importance. **f** Fecal VIP plot indicating the most discriminating bacteria in descending order of importance. Statistical comparisons between genotype and diet were determined by ANOVA, followed by Tukey’s post hoc test. PLS-DA and VIP scores were assessed using MetaboAnalyst 3.0. *Ellipses* represent 95 % confidence intervals for each individual group on PLS-DA plots with *Q* ^2^ and *R* ^2^ values being used to assess the robustness of the model and the amount of variation represented by the principal components, respectively. VIP scores reflect the degree of importance of a bacteria, with values >1.0 seen as driving the calculated discrimination. All data were collected using qRT-PCR and are presented as mean 16S rRNA gene copies/mg of corresponding tissue ± SEM (CC, B6-chow; CK, B6-ketogenic; BC, BTBR-chow; and BK, BTBR-ketogenic; *n* = 11, 10, 15, and 10, respectively). *p* < 0.01 if values do not share a superscript letter. An adapted version of this table has been previously published. Reprinted with permission of the American Chemical Society, Copyright 2016

Our results identified distinct differences in animal mass between control and BTBR animals (Table 1). Further differences between genotype were noted following 16S rRNA microbial profiling of cecal and fecal samples. Examination of the data employing multivariate analysis discerned that our control (B6) and BTBR mice had dissimilar cecal and fecal microbial profiles (Fig. 1a–d and Table 2). The top three metabolites responsible for driving the separation of groups included *A. muciniphila*, *Methanobrevibacter* spp., and *Roseburia* spp. in the cecal samples and *A. muciniphila*, Enterobacteriaceae, and *Lactobacillus* in the fecal samples.

**Table 1:** Animal characteristics

	B6		BTBR	
	Chow	Ketogenic	Chow	Ketogenic
Mass (g)	18.4 ± 0.8^a^	10.5 ± 0.3^b^	28.6 ± 1.3^c^	15.9 ± 1.0^a^
Blood glucose (mmol/L)	10.4 ± 0.6^a^	4.3 ± 0.5^b^	7.8 ± 0.3^c^	3.7 ± 0.5^b^
Blood ketones (mmol/L)	0.9 ± 0.1^a^	5.1 ± 0.8^b^	0.9 ± 0.1^a^	5.1 ± 0.8^b^

Age-matched B6 and BTBR mice were sacrificed following 10–14 days of either chow or ketogenic feeding. All characteristics were assessed prior to sacrifice. Data are mean ± SEM (B6-chow, B6-ketogenic, BTBR-chow, and BTBR-ketogenic; *n* = 11, 10, 15, and 10, respectively). *p* < 0.01 if superscripts do not share a letter (^a^, ^b^, and ^c^)

**Table 2:** Microbiota composition in cecal and fecal samples from B6 and BTBR mice following chow or ketogenic feeding

		B6		BTBR	
		Chow	Ketogenic	Chow	Ketogenic
Cecal microbes (×1000)	*Akkermansia muciniphila*	1.1 ± 0.1^a^	0.3 ± 0.04^b^	1011 ± 153^c^	0.2 ± 0.02^b^
	*Bacteroides/Prevotella* spp.	7028 ± 611^ab^	5044 ± 1114^a^	9233 ± 1211^b^	709 ± 317^c^
	*Bifidobacterium* spp.	6132 ± 1071^a^	17.6 ± 4.8^b^	381 ± 95^c^	5.6 ± 0.9^d^
	*Clostridium* cluster I	24.6 ± 2.2^a^	9.3 ± 1.7^b^	13.9 ± 1.7^c^	6.2 ± 0.7^b^
	*Clostridium* cluster XI	1.4 ± 0.1^a^	3.7 ± 0.8^b^	1.0 ± 0.09^c^	2.7 ± 0.3^b^
	*Clostridium coccoides*	53,223 ± 4428^a^	17,128 ± 4253^b^	46,568 ± 3885^a^	11,157 ± 2451^b^
	*Clostridium leptum*	4959 ± 811^ab^	2668 ± 690^ac^	6150 ± 612^b^	1570 ± 281^c^
	Enterobacteriaceae	257 ± 42^a^	70 ± 12^b^	77 ± 7.9^b^	36 ± 4.7^c^
	Total Firmicutes	61,819 ± 4641^a^	19,874 ± 4290^b^	57,015 ± 4086^a^	12,795 ± 2602^b^
	*Lactobacillus* spp.	3609 ± 1078^a^	65 ± 31^b^	4279 ± 790^a^	59 ± 36^b^
	*Methanobrevibacter* spp.	2.6 ± 0.5^a^	3.4 ± 0.2^b^	10 ± 0.9^c^	2.9 ± 0.4^ab^
	*Roseburia* spp.	2.4 ± 0.3^a^	0.7 ± 0.08^b^	3.3 ± 0.3^a^	0.5 ± 0.1^b^
	Firmicutes/Bacteroidetes ratio	9.4 ± 1.0^a^	4.6 ± 1.2^a^	6.9 ± 0.7^a^	32.5 ± 5.8^b^
Fecal microbes (×1000)	*Akkermansia muciniphila*	0.2 ± 0.02^ab^	0.3 ± 0.06^ac^	1392 ± 223^d^	0.2 ± 0.03^bc^
	*Bacteroides/Prevotella* spp.	11,734 ± 1128^a^	31,189 ± 6810^b^	22,562 ± 3294^b^	3654 ± 903^c^
	*Bifidobacterium* spp.	10,414 ± 999^a^	120 ± 27^b^	951 ± 229^c^	22.6 ± 5.2^d^
	*Clostridium* cluster I	5.6 ± 0.4^a^	12.7 ± 2.0^bc^	18.0 ± 2.7^bd^	15.3 ± 2.4^cd^
	*Clostridium* cluster XI	11.6 ± 1.1^ab^	8.2 ± 1.4^ac^	4.4 ± 1.3^d^	10.6 ± 1.5^bc^
	*Clostridium coccoides*	26,467 ± 4814^a^	20,859 ± 3938^a^	36,587 ± 4428^a^	37,019 ± 7867^a^
	*Clostridium leptum*	2879 ± 613^ab^	3015 ± 594^a^	3956 ± 364^bc^	7245 ± 1508^c^
	Enterobacteriaceae	253 ± 25^ab^	633 ± 161^ac^	116 ± 9.5^d^	419 ± 112^bc^
	Total Firmicutes	37,399 ± 5318^a^	24,121 ± 3998^b^	49,118 ± 4672^a^	44,782 ± 7907^a^
	*Lactobacillus* spp.	8031 ± 1573^a^	225 ± 80^b^	8548 ± 1535^a^	492 ± 134^b^
	*Methanobrevibacter* spp.	9.0 ± 0.7^a^	0.7 ± 0.1^b^	5.7 ± 1.9^c^	0.8 ± 0.1^b^
	*Roseburia* spp.	5.3 ± 0.6^a^	1.3 ± 0.2^b^	5.3 ± 0.9^a^	0.9 ± 0.1^b^
	Firmicutes/Bacteroidetes ratio	3.5 ± 0.7^a^	1.2 ± 0.3^a^	2.4 ± 0.2^a^	13 ± 1.8^b^

Total Firmicutes (*Clostridium coccoides*, *Clostridium leptum*, *Clostridium* clusters XI and I, *Roseburia* spp., and *Lactobacillus* spp.) and Bacteroidetes (*Bacteroides/Prevotella* spp.). Data are mean 16S rRNA gene copies/mg cecal or fecal matter ± SEM (*n* = 11, 10, 15, and 10 respectively). *p* < 0.05 if values do not share a superscript letter

Cecal and fecal tissues were both assessed in the present study as research indicates that microbial abundance and diversity are directly related to the physiological role of each segment of the GI tract [17]. Supporting previous work, our data shows an increase in total microbial content further down the GI tract and an increase in the obligate anaerobes Bacteroidetes (*Bacteroides*/*Prevotella* spp.) in fecal tissues [17]. However, with the exception of Enterobacteriaceae, directional changes occurring with the KD were generally conserved between these two sampling sites.

Examination of our data show several alterations in fecal gut microbiota of BTBR mice that are observed in patients with ASD, including elevated *Clostridium* cluster XI [18], decreased Firmicutes (*Clostridium coccoides*, *Clostridium leptum*, *Clostridium* clusters XI and I, *Roseburia* spp., and *Lactobacillus* spp.) [2] and increased Bacteroidetes [1]. Taken together, our results indicate that the BTBR mouse model may provide insight into the role of gut-brain interactions in ASD and that it may be useful in testing the impact of interventions such as pre- and probiotic administration on the disease.

Results of both cecal and fecal analysis showed a significant decline in total bacterial content upon implementation of the KD, in both animal genotypes (Fig. 1a, b). Interestingly, the KD decreased total cecal and fecal microbes in BTBR animals by a mean of 78 and 28 %, respectively. These are explained by the gut microbiota’s primary responsibility to degrade undigested carbohydrates, which are substantially diminished in the KD [19]. Consistent with this, short-term administration of vancomycin, a broad-spectrum oral antibiotic, has been reported to improve behavioral symptoms of ASD in young boys [20]. As vancomycin is unable to be absorbed and has no interaction with the central nervous system, the resulting behavioral improvements are thought to involve gut microbiome-drug interactions [21].

Although the gut microbiome is comprised of hundreds of discernible species, approximately 90 % of measured 16S rRNA sequences belong to the Firmicutes or Bacteroidetes phyla [19]. While estimations of complete microbial diversity remain a limitation of 16S profiling, a targeted approach was employed to identify commonly abundant microbial species. Interestingly, the decrease in Firmicutes and increase in Bacteroidetes across both cecal and fecal matter were mitigated in BTBR animals fed a KD (Table 2). The Firmicutes phylum is comprised of several classes of gram-positive bacteria which include Clostridia [22]. Clostridia can be further divided into approximately 20 clusters including the abundant *C. coccoides* and *C. leptum* [23]. *C. coccoides* and *C. leptum* are saccharolytic bacteria that generate short-chain fatty acids (SCFAs) [23]. Recent research suggests that the SCFAs butyrate and propionate actively communicate with the brain [24]. Our data shows a relative two- to threefold increase in the known SCFA generating *C. coccoides* and *C. leptum* populations, respectively, when cecal and fecal samples of BTBR-ketogenic animals are compared to all other groups (Table 2). The ability to alter the ASD microbial phenotype in the BTBR mouse through dietary manipulation may elucidate the mechanism of action connecting KD and its capacity to improve ASD behavioral symptoms.

A variety of neurological disorders, including ASD, is associated with various GI symptoms, including abdominal pain, inflammation, and impaired gastric motility [23]. These symptoms are thought to be partially attributed to the maintenance of the mucosa, a multi-layered tissue comprising the innermost layer of the GI tract. Compromising the integrity of this physical barrier results in intestinal permeability and has been highlighted in clinical cases of ASD [9, 25]. The mucin protein family is the predominant source of proteins contributing to mucus secretions from the epithelial layer of the mucosa [26]. Within the GI lumen resides the mucin-degrading bacterium *A. muciniphila*—the predominant representative of the Verrucomicrobia phylum within the GI tract [27]. Responsible for maintaining homeostasis of mucus secretions, research suggests that diminished gut microbial diversity [28], mucosal infiltration [27], and ASD [1, 2] are linked to increased *A. muciniphila.* Our results indicate that BTBR animals fed a chow diet have significantly elevated *A. muciniphila* in both cecal and fecal matter (Table 2). The source or underlying cause of this elevation is not known. However, the ketogenic diet resulted in a normalization of *A. muciniphila* bacteria in BTBR mice, resulting in similar levels to those found in control (B6), chow-fed animals. In fact, this bacterial species was the primary bacteria driving the calculated multivariate discrimination in both our cecal and fecal samples (Fig. 1e, f).

In summary, this study demonstrates that KD consumption triggers gut microbiota remodeling in an animal model of ASD. These findings may provide insight into the therapeutic potential of KD manipulation by influencing gut microbial composition. Due to the paucity of research examining the role of the microbiome in KD therapy, we suggest further investigations into the complex interplay between the gut microbiome and the brain relevant to ASD and other neurological disorders.

## Additional file

Additional file 1: Table S1. Gut microbial group-specific primers and genomic DNA standards for qRT-PCR. (DOC 48 kb)

## Abbreviations

ANOVA: Analysis of variance
ASD: Autism spectrum disorder
CH: Control diet
KD: Ketogenic diet
PLS-DA: Partial least squares discriminant analysis
SCFAs: Short-chain fatty acids
VIP: Variable importance of projection
