# Supplementary material for: Ketogenic diet modifies the gut microbiota in a murine model of autism spectrum disorder
Source: Mol Autism. 2016 Sep 1;7(1):37. doi: 10.1186/s13229-016-0099-3 (PMC5009541; doi:10.1186/s13229-016-0099-3)
Supplement: Additional file 1: Table S1. — Gut microbial group-specific primers and genomic DNA standards for qRT-PCR. (DOC 48 kb) [file 13229_2016_99_MOESM1_ESM.doc]

**Supplementary Table 1** Gut microbial group specific primers and genomic DNA standards for qRT-PCR

| **Microbial Group** | **Primer Sequence, 5’-3’**  **(Forward, F and Reverse, R)** | **Genomic DNA Standard** | **Reference** |
| --- | --- | --- | --- |
| **Total bacteria** | F: ACTCCTACGGGAGGCAGC  R: CCGTMTTACCGCGGCTGCTGGCA | *Escherichia coli* | Amann, Krumholz, & Stahl, 1990; W.T. Liu, Mirzabekov, & Stahl, 2001 |
| **Firmicutes** |  |  |  |
| ***Clostridium coccoides* (cluster XIV)** | F: ACTCCTACGGGAGGCAGC  R: GCTTCTTAGTCARGTACCG | *Ruminococcus productus* | Amann, Krumholz, & Stahl, 1990; Franks et al., 1998 |
| ***Clostridium leptum* (cluster IV)** | F: GCACAAGCAGTGGAGT  R: CTTCCTCCGTTTGTCAA | *Clostridium leptum* | Matsuki, Watanabe, Fujimoto, Takada, & Tanaka, 2004 |
| ***Clostridium* cluster I** | F: ATGCAAGTCGAGCGAKG  R: TATGCGGTATTAATCTYCCTTT | *Clostridium perfringens* | Rinttila, Kassinen, Malinen, Krogius, & Palva, 2004 |
| ***Clostridium* cluster XI** | F: ACGCTACTTGAGGAGGA  R: GAGCCGTAGCCTTTCACT | *Clostridium difficile* | Song, Liu, & Finegold, 2004 |
| ***Lactobacillus* spp.** | F: GAGGCAGCAGTAGGGAATCTTC  R: GGCCAGTTACTACCTCTATCCTTCTTC | *Lactobacillus jensonii* | Delroisse et al., 2008 |
| ***Roseburia* spp.** | F: TACTGCATTGGAAACTGTCG  R: CGGCACCGAAGAGCAAT | *Roseburia hominis* | Larsen et al., 2010 |
| **Bacteroidetes** |  |  |  |
| ***Bacteroides/Prevotella* spp.** | F: TCCTACGGGAGGCAGCAGT  R: CAATCGGAGTTCTTCGTG | *Bacteroides thetaiotaomicron* | Bernhard & Field, 2000; Nadkarni, Martin, Jacques, & Hunter, 2002 |
| **Actinobacteria** |  |  |  |
| ***Bifidobacterium* spp.** | F: CGCGTCYGGTGTGAAAG  R: CCCCACATCCAGCATCCA | *Bifidobacterium adolescentis* | Delroisse et al., 2008 |
| **Archaea** |  |  |  |
| ***Methanobrevibacter* spp.** | F: CTCACCGTCAGAATCGTTCCAGTC  R: ACTTGAGATCGGGAGAGGTTAGAGG | *Methanobrevibacter smithii* | Bomhof et al., 2014 |
| **Proteobacteria** |  |  |  |
| **Enterobacteriaceae** | F: CATTGACGTTACCCGCAGAAGC  R: CTCTACGAGACTCAAGCTTGC | *Escherichia coli* | Bartosch, Fite,  Macfarlane, & McMurdo, 2004 |
| **Verrucomicrobia** |  |  |  |
| ***Akkermansia muciniphila*** | F: TCTTCGGAGGCGTTACACAG  R: AGTTGATCTGGGCAGTCTCG | *Akkermansia muciniphila* | Beacon Designer 3.0 |
